# Prevalence of diabetes mellitus in long-term cancer survivors treated with craniospinal irradiation

**DOI:** 10.1210/jendso/bvag116

**Published:** 2026-06-13

**Authors:** Franziska Richter, Cassandra Runow, Denise Obrecht-Sturm, Lea L Kronziel, Stefan Rutkowski, Desiree Grabow, Christian Denzer, Thorsten Langer, Judith Gebauer

**Affiliations:** Department of Pediatric Oncology and Hematology, University Medical Center Schleswig-Holstein, 23562 Luebeck, Germany; Department of Pediatric Oncology and Hematology, University Medical Center Schleswig-Holstein, 23562 Luebeck, Germany; Department of Hematology and Oncology, University Medical Center Schleswig-Holstein, 23562 Luebeck, Germany; Department of Pediatric Oncology and Hematology, University Medical Center Hamburg-Eppendorf, 20246 Hamburg, Germany; Institute of Medical Biometry and Statistics, University of Luebeck, 23562 Luebeck, Germany; Department of Pediatric Oncology and Hematology, University Medical Center Hamburg-Eppendorf, 20246 Hamburg, Germany; Division of Childhood Cancer Epidemiology, German Childhood Cancer Registry, Institute of Medical Biostatistics, Epidemiology and Informatics (IMBEI), University Medical Center of the Johannes Gutenberg University Mainz, 55131 Mainz, Germany; Division of Pediatric Endocrinology and Diabetes, Department of Pediatrics and Adolescent Medicine, University Medical Center, 89075 Ulm, Germany; Department of Pediatric Oncology and Hematology, University Medical Center Schleswig-Holstein, 23562 Luebeck, Germany; Department of Hematology and Oncology, University Medical Center Schleswig-Holstein, 23562 Luebeck, Germany; Department of Oncology and University Cancer Center Leipzig, 04103 Leipzig, Germany

**Keywords:** diabetes mellitus, childhood cancer survivors, medulloblastoma, irradiation, late effects

## Abstract

**Background:**

Childhood, adolescent, and young adult cancer survivors (CAYA) are at an increased risk for metabolic disorders such as diabetes mellitus. Although this risk has been well-documented for leukemia survivors and those exposed to abdominal radiotherapy, the metabolic risk profile for brain tumor survivors remains largely unexplored.

**Methods:**

This study assessed the prevalence of diabetes and dysglycemia in 2 CAYA survivor cohorts. The first included medulloblastoma and ependymoma survivors (HIT-MED), with data collected via self-reported online questionnaires. The second (CLI) was a retrospective analysis of an unselected cohort from our late effects clinic. In this cohort, dysglycemia was defined using hemoglobin A1c measurements alone, without additional glucose-based diagnostic criteria.

**Results:**

In the HIT-MED group, 37% (197/527) participated in an online-based questionnaire, revealing a diabetes mellitus prevalence of 3.05%. A total of 122 were male and 75 female, mean age was 27.1 ± 4.5 years (range 19-41). In comparison to an age-matched general population, there was no significant difference (*P* = .468). Among survivors in the CLI group who received craniospinal irradiation (mean age 38 years at assessment), 34.6% (9/26) exhibited dysglycemia, including 7.7% (2/26) with diabetes mellitus and 26.9% (7/26) with prediabetic glucose levels. This represented the second highest prevalence of impaired glucose regulation among all irradiation subgroups.

**Conclusion:**

CAYA brain tumor survivors exposed to craniospinal radiotherapy have an elevated risk of dysglycemia compared to other survivor cohorts. These findings support the need for structured, risk-adapted long-term metabolic monitoring to enable early intervention and reduce long-term morbidity.

Progress in childhood cancer treatment has, over the past decades, improved long-term survival, which currently exceeds 84% [1]. However, many children, adolescents and young adults (CAYA) cancer survivors experience chronic health limitations and the cumulative incidence of these conditions increases with time elapsed since the end of therapy. Depending on the type and intensity of cancer therapy, late effects can impact various organ systems, ranging from mild to severe and sometimes life-threatening conditions [2-4].

In this context, it has been recognized that the risk of developing diabetes mellitus (DM) later in life is higher among CAYA cancer survivors compared to the general population [5, 6]. Particularly, radiation therapy has been identified as the major risk factor for the development of DM during follow-up [7]. In a retrospective, questionnaire-based study, French researchers determined a relative risk of 11.5 for DM in irradiated compared to nonirradiated patients, whereas no association with chemotherapy was established [8]. This cohort mainly included former patients with nephroblastoma, neuroblastoma, lymphoma, and sarcoma, with a smaller subset of patients with brain tumors also examined [8]. A total of 18% of study participants reported receiving insulin monotherapy, suggesting a significant proportion of patients with type 1 DM in this cohort.

In a North American study, 40 patients who underwent abdominal radiation were investigated to determine potential pathomechanisms resulting in increased DM risk after radiation exposure [9]. Nine patients exhibited glucose homeostasis disorders, 1 of whom presented with overt DM; the remaining patients either had impaired fasting glucose or impaired glucose tolerance. Patients affected by prediabetes were more insulin resistant than nonaffected individuals.

Lee et al (2020) investigated the occurrence of DM in leukemia survivors treated with allogenic hematopoietic cell transplantation [10]. A 10-year cumulative incidence of 5% (95% CI, 3-7) for DM was observed in this cohort, based on data derived from 826 patients. The comparison between exposure to chemotherapy only or concomitant total body irradiation (TBI) demonstrated a higher risk for DM after TBI.

In summary, the underlying cause for the increased risk of DM following cancer therapy in childhood is not conclusively determined, although most authors attribute it to a more frequent development of type 2 DM, and consequently a higher risk for insulin resistance. Abdominal radiotherapy may result in a more rapid loss of β-cell capacity or function. However, there is a lack of systematically collected data for a better differentiation of DM types in cancer survivors. Furthermore, it is not yet certain to what extent the risk of DM increases linearly with increasing radiation dose or if a plateau occurs at a dose of 20 to 29 Gy (in the area of the pancreatic tail) [11].

Most studies on this topic have so far been conducted on survivors of intra-abdominal tumors (especially neuro- and nephroblastoma survivors), recognizing abdominal radiation therapy exposure as a major contributing factor, as well as an increased risk demonstrated for patients with hematologic diseases [8, 9, 12-14]. Former brain tumor patients have been included in few studies, but further information, particularly regarding histology and tumor therapy has not been provided. Accordingly, DM in survivors treated with craniospinal irradiation (CSI), which involves both cranial and abdominal radiation therapy at doses exceeding those of TBI by 2- to 3-fold, remains unclear.

As DM and prediabetes constitute major cardiovascular risk factor and contribute to an increased risk for further health impairment, the identification of risk groups is crucial to offer risk adapted long-term follow-up (LTFU) care [6, 15, 16].

In the present study, the prevalence and respective subtypes of DM in long-term survivors treated with radiotherapy, surgery, and chemotherapy will be assessed with an online-based questionnaire survey and with a retrospective analysis of clinical data including prediabetic state. Furthermore, differences in DM prevalence between CAYA cancer survivors undergoing different radiotherapy regimens will be investigated. The results of this study will help to develop targeted and risk-adapted recommendations for early detection and timely treatment of DM in brain tumor survivors through focused clinical and laboratory investigations.

## Materials and methods

The study is based on 2 different cohorts, HIT-MED and CLI, which were analyzed separately.

### Study population HIT-MED group

An online-based questionnaire survey of all long-term survivors of medulloblastoma or ependymoma treated within the framework of the Society for Pediatric Oncology and Hematology HIT-MED clinical studies, as well as patients from the Interim Register and also HIT-MED Register were included, and registered by the HIT-MED trial center at the University Medical Center Hamburg-Eppendorf has been performed assessing the prevalence, respective subtype, and treatment of DM, as well as the presence of other cardiovascular risk factors. The link to the online survey was provided from January to March 2023. A total of 527 patients were contacted; 197 completed the study. Diabetes risk in brain tumor survivors was compared to the general population (reference data [17]). Additionally, the prevalence of cardiovascular risk factors in CAYA cancer survivors affected by DM was determined.

### Inclusion criteria HIT-MED group

Inclusion criteria were diagnosis and therapy of medulloblastoma or ependymoma, age at least 18 years at study entry, at least 5 years since cancer treatment, and available basic variable set including therapy data at the HIT-MED trial center.

### Exclusion criteria HIT-MED group

Not registered to the German Childhood Cancer Registry

Based on these criteria, a total of 527 CAYA cancer survivors were identified by the HIT-MED trial center. All survivors who had agreed to be contacted were invited to participate in the study through the German Childhood Cancer Registry. Participants received a link to log in to SosciSurvey, a platform for online surveys, along with their personal participant identification number. Study participation was reimbursed with a gift voucher of 10 euros after successful completion of the questionnaires.

All data were electronically documented on the platform and after study completion, were transferred to a statistical analysis program. Patients’ characteristics were obtained through data extraction from the HIT-MED database and combined with the questionnaire results for the final analysis.

### Study population CLI group

This part of the study included LTFU patients treated in our late effects clinic at University Medical Center Schleswig-Holstein, Luebeck, between March 2014 and September 2024.

### Inclusion criteria CLI group

Inclusion criteria were the participation in the LTFU program at the University Medical Center Schleswig-Holstein (described in detail in a previous publication [18]), diagnosed with cancer at the age of 21 or younger and available clinical data including hemoglobin A1c (HbA1c) from at least 1 time point during March 2014 and September 2024.

### Exclusion criteria CLI group

Patients were excluded if they were under active cancer treatment (eg, or subsequent neoplasms), if they were minors at the time of their last follow-up visit, or if they died during the study period.

A total of 457 patient records were reviewed. Based on the defined criteria, 350 patients were included in the analysis. Their clinical data were extracted from hospital records obtained during routine follow-up visits. These data included demographic and clinical variables such as age at follow-up, sex, age at cancer diagnosis, type of cancer, treatment exposures, anthropometric measurements, laboratory parameters, and cardiovascular risk factors. We determined random blood glucose levels and HbA1c values of all patients. Every patient in our long-term follow-up care clinic undergoes an HbA1c measurement as part of our routine assessment, regardless of any indications of dysglycemia. Patients with DM were classified based on their preexisting diagnosis, whereas the remaining patients were categorized based on their HbA1c values. Prediabetes was defined according to the American Diabetes Association as an HbA1c of 5.7% to 6.4%, whereas regular metabolic state was defined as an HbA1c ≤ 5.6% [19]. For patients with an HbA1c of 5.7% or higher, we offered an oral glucose tolerance test (OGTT) to allow us to at least exclude manifest diabetes in these individuals. After performing the OGTT, insulin sensitivity was calculated based on the Matsuda index [20, 21].

To assess the effect of CSI on metabolic health, patients were categorized into 5 treatment groups according to the type of radiotherapy received: TBI, whole cranial irradiation, CSI, abdominal irradiation, and no or other forms of irradiation. Furthermore, additional therapeutic exposures such as chemotherapy regimens, systemic glucocorticoid therapy as part of antineoplastic treatment, surgical treatments and stem cell transplantation were documented and included in the statistical analyses. Applications of glucocorticoids solely as antiemetics or for the management of intracranial pressure were excluded from this analysis.

The data collected during clinical follow-up visits were electronically documented in hospital records and later extracted for statistical analysis.

### Statistical analysis

Descriptive statistics were calculated for continuous variables in the HIT-MED group, including median and range. The absolute and relative number in each category is shown for categorical variables.

The prevalence of patients with diabetes mellitus in the study population was estimated and reported with a 95% CI. A statistical test for a deviating prevalence was performed using the DEGS1 study as a comparison cohort [17]. The sex-specific prevalence stated therein was averaged (0.9% male, 3.7% female). An exact binomial test with a 5% significance level was performed. The *P* value and the 95% CI are reported.

No further calculations in the subgroup of patients diagnosed with DM were carried out because of the small number of patients in this subgroup.

The programming language R with version 4.3.3 is used for the entire analysis of the data.

The statistical analysis for the CLI group were performed using Jamovi (Version 2.6.44.0), which is a free and open statistical platform built on the R programming language. For descriptive statistics, the median and range for continuous variables and absolute and relative frequencies for categorical variables are presented. *P* value calculation were conducted using the programming language R with version 4.5.1.

To determine the association between the type of radiotherapy and the metabolic status, a Fisher's test was performed using Monte Carlo simulation to approximate the *P* value, as both variables were categorical. The analysis included the 5 different radiotherapy groups and their relationship to the HbA1c-based metabolic classification (normal, prediabetic, diabetic). To specifically assess the association between craniospinal irradiation and dysglycemia, a Fisher's exact test was performed comparing the CSI group vs all other radiation groups combined. The *P* value is reported.

HbA1c and random glucose levels were compared across the 5 radiation groups using ANOVA. To control for potential confounding effects of body mass index (BMI), an analysis of covariance (ANCOVA) was subsequently conducted. In addition, potential confounding by steroid exposure was evaluated using a logistic regression model, with dysglycemia as the outcome variable and radiation group and steroid use included as covariates.

### Ethical approval

The study was approved by the ethics committee of the University of Luebeck (22-137 and 2022-647) and conducted in accordance with the Helsinki Declaration of 1975. Informed consent was obtained from all participants included in the study.

## Results

### Study population HIT-MED group

Overall, 197 CAYA cancer survivors (122 males and 75 females) participated in the online survey. Six patients reported a diagnosis of DM resulting in a prevalence of 3.05% (95% CI, 0.65-5.44). Compared to the German general population (DEGS1 study [17]), no significant difference in diabetes prevalence was found (*P* = .468; 95% CI, 0.01-0.07).

Patient characteristics of the total study cohort and the subgroup with DM are presented in Table 1. The total study cohort had a median BMI of 23.42 kg/m^2^ (range 16.51-52.47 kg/m^2^), whereas the DM subgroup had a median BMI of 36.86 kg/m^2^ (range 30.01-52.47 kg/m^2^). Regarding weight development, 64 study participants (32.5%) reported that they weighed the same as before their cancer diagnosis; 9 (4.6%) lost weight since being diagnosed with cancer and 100 (50.76%) stated that they had gained weight since their cancer diagnosis. Five (83%) patients in the DM subgroup also gained weight. A total of 24 (12.18%) participants gave no information about the development of their body weight. Of the patients who reported that their body weight had changed significantly since being diagnosed with cancer, 37 (18.78%) had tried to gain weight, whereas 25 (12.69%) stated that they had tried to lose weight to achieve a normal body weight. A total of 129 (65.48%) study participants considered this question was not relevant. We conducted a nonresponder analysis to examine differences between participants and nonparticipants in the HIT-MED group. No relevant differences were found for gender, age at inclusion, decade of diagnosis, or type of brain tumor (ependymoma or medulloblastoma). However, participants were slightly older at the time of brain tumor diagnosis than nonresponders.

**Table 1:** Patient characteristics of the HIT-MED group in comparison to the subgroup with DM

Variable	Total cohort (n = 197)	Subcohort with diagnosis of DM (n = 6)
Sex		
Male	122 (61.9%)	2 (33.3%)
Female	75 (38.1%)	4 (66.7%)
Median age at cancer diagnosis, y		
Median (range)	9.06 (0.5-17.6)	10.4 (1.7-12.2)
Time since diagnosis, y		
Median (range)	18.7 (8.3-31.7)	17.5 (13.3-21.5)
Entity		
Medulloblastoma	138 (70.1%)	3 (50%)
Ependymoma	59 (30%)	3 (50%)
**Cancer therapy**		
Chemotherapy		
Yes	165 (83.8%)	4 (66.7%)
No	20 (10.2%)	1 (16.7%)
Unknown	12 (6.1%)	1 (16.7%)
Surgery		
Yes	197 (100%)	6 (100%)
Radiotherapy		
No RT data available	14 (7.1%)	0 (0%)
RT as primary therapy	179 (90.9%)	6 (100%)
RT treatment as relapse treatment	4 (2.0%)	0 (0%)
**Risk factors**		
Smoking		
Yes	15 (7.61%)	0 (0%)
No	176 (89.3%)	6 (100%)
Not answered	6 (3.1%)	0 (0%)
Hypertension		
Yes	8 (4.1%)	3 (50%)
No	183 (92.9%)	3 (50%)
Not answered	6 (3.1%)	0 (0%)
Hyperlipidemia		
Yes	24 (12.2%)	5 (83.3%)
No	167 (84.8%)	1 (16.7%)
Not answered	6 (3.1%)	0 (0%)
**Cardiovascular disease**		
Heart attack or coronary artery disease		
Yes	0 (0%)	0 (0%)
No	191 (97%)	6 (100%)
Not answered	6 (3.1%)	0 (0%)
Stroke		
Yes	7 (3.6%)	1 (16.7%)
No	184 (93.4%)	5 (83.3%)
Not answered	6 (3.1%)	0 (0%)

Abbreviations: DM, diabetes mellitus; RT, radiotherapy.

### DM subgroup

Six CAYA cancer survivors developed DM. One person had type 1 DM, 4 had type 2 DM, and 1 patient reported having another type of DM. Median age of these patients was 23 years (range 15-32). Four patients (67%) reported taking metformin; only 1 individual reported being treated with insulin and 3 (50%) reported not taking DM-specific medications. Three patients stated that first-degree relatives also had DM, 1 person reported that grandparents had DM, and 2 did not answer this question.

### Study population CLI group

A total of 350 CAYA survivors (158 male and 192 female) were included in this analysis. Fifty-three (15.2%) patients had brain tumors, 110 (31.4%) had leukemias, 75 (21.4%) had lymphomas, 50 (14.3%) had sarcomas, 49 (14.0%) had embryonal tumors, and 13 (3.7%) had rare tumors. Regarding oncological treatment, 301 (86.0%) patients received chemotherapy, including 163 (46.6%) who received steroids as part of their therapeutic regimen. A total of 179 (51.1%) were treated with radiotherapy, 142 (40.6%) underwent surgery, and 42 (12.0%) received a stem cell transplantation.

All relevant clinical data and various cardiovascular risk factors were documented in the different radiotherapy groups and are summarized in Table 2.

**Table 2:** Patient characteristics in the CLI group

	TBI (n = 13)		Whole cranial radiation (n = 75)		Craniospinal radiation (n = 26)		Abdominal radiation (n = 46)		No or other radiation (n = 190)	
Age at recent visit, y Median + range	28.0	18-41	30.0	18-65	29.5	20-47	37.0	19-57	28.0	18-59
Sex										
Female	10	76.9%	42	56.0%	10	38.5%	35	76.1%	105	55.3%
Male	3	23.1%	33	44.0%	16	61.5%	11	23.9%	85	44.7%
Age at diagnosis, y Median + range	7.0	2-17	7.0	0-21	8.0	1-21	12.0	0-19	12.0	0-21
Diagnosis										
Brain tumors	0		13	17.3%	21	80.8%	0		19	10.0%
Leukemias	13	100%	40	53.3%	3	11.5%	1	2.2%	53	27.9%
Lymphomas	0		3	4.0%	0		24	52.2%	48	25.3%
Sarcomas	0		9	12.0%	1	3.8%	10	21.7%	30	15.8%
Embryonal tumors	0		8	10.7%	1	3.8%	11	23.9%	29	15.2%
Rare tumors	0		2	2.7%	0		0		11	5.8%
Therapy										
Chemotherapy	13	100%	68	90.7%	25	96.2%	45	97.8%	150	78.9%
Systemic steroids	13	100%	32	42.7%	3	11.5%	25	54.3%	90	47.4%
Radiation	13	100%	75	100%	26	100%	46	100%	19	10.0%
Surgery	2	15.4%	23	30.7%	18	69.2%	21	45.7%	78	41.1%
Stem cell transplantation	13	100%	6	8.0%	0		5	10.9%	18	9.5%
Cardiovascular risk factors										
BMI in kg/m^2^ Median + range	20.0	14.8-36.6	25.0	17.6-43.0	25.5	16.8-31.4	22.7	16.4-35.5	24.0	15.0-54.7
Waist circumference in cm Median + range	76.0	62-103	88.0	62-129	84.5	63-107	79.0	57-120	81.0	61-150
High blood pressure	2/13	15.4%	7/75	9.3%	2/26	7.7%	7/46	15.2%	14/190	7.4%
Hypercholesterolemia	7/13	53.9%	36/75	48.0%	10/26	38.5%	18/46	39.1%	49/190	25.8%
Stroke	0		3/75	4.0%	0		0		1/190	0.5%
Heart attack	0		0		0		1/46	2.2%	0	
Current nicotine use	1/13	7.7%	9/75	12.0%	2/26	7.7%	3/46	6.6%	32/190	16.8%

Abbreviations: BMI, body mass index; TBI, total body irradiation.

**Figure 1:**
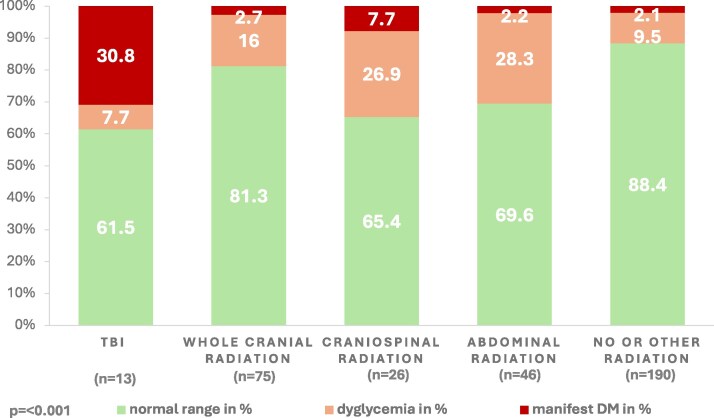
Diabetic metabolic states in different radiotherapy groups within the CLI group, displayed as relative proportions.

The prevalence of DM calculated using the Fisher test with Monte Carlo simulation varied significantly among the 5 radiotherapy groups (*P* < .001). However, a separate comparison of the CSI group with all remaining radiotherapy groups using an exact Fisher's test showed no significant difference in dysglycemia prevalence (*P* = .07096). The distribution of states of glucose homeostasis across different radiotherapy groups is illustrated in Fig. 1. The highest proportion of patients with manifest DM was discovered in the TBI group, where 4 of 13 (30.8%) patients were affected, and 1 further (7.7%) patient was classified with prediabetes. In the CSI group, 2 of 26 (7.7%) patients had DM, whereas 7 (26.9%) presented prediabetic HbA1c levels. Similarly, in the abdominal irradiation group, 1 of 46 (2.2%) patients had DM, whereas 13 (28.3%) had prediabetic HbA1c values. The lowest prevalence of DM was found in the no or other forms of irradiation group, where 4 of 190 (2.1%) patients had a manifest DM and 18 (9.5%) had prediabetic levels of HbA1c values.

Among patients with DM, additional subgroup analyses were performed. Relevant characteristics, including demographic characteristics, anthropometric measurements, and details on DM treatment are presented in Table 3.

**Table 3:** Characteristics of the patients with DM in the CLI group

	TBI (n = 13)		Whole cranial radiation (n = 75)		Craniospinal radiation (n = 26)		Abdominal radiation (n = 46)		No or other radiation (n = 190)	
Patients with DM	4/13	30.8%	2/75	2.7%	2/26	7.7%	1/46	2.2%	4/190	2.1%
Age at recent visit, y Median + range	29.0	18-41	40.0	32-48	38.0	29-47	39.0	39-39	46.0	35-55
Sex										
Female	4	100%	1	50.0%	2	100%	1	100%	0	0%
Male	0	0%	1	50.0%	0	0%	0	0%	4	100%
HbA1c in % Median + range	6.4	6.3-6.5	6.9	6.7-7.0	6.8	6.4-7.1	6.0		6.6	6.0-7.2
Random blood glucose levels, mg/dL Median + range	118.5	96-143	134.0	96-172	84.0	79-89	90.0		106.0	49-129
DM therapy										
Dietary	3		0		1		0		2	
Metformin	1		1		0		0		0	
GLP-1 analogs	0		0		1		1		0	
Insulin	0		2		0		0		2	
Cardiovascular risk factors										
BMI, kg/m^2^ Median + range	20.1	19.8-36.6	30.3	28.3-32.3	26.9	25.5-28.2	24.1	24.1-24.1	24.4	23.3-41.6
Waist circumference, cm Median + range	82.0	73-103	103.0	102-104	85.0	85-85	88.0	88-88	92.0	80-119
High blood pressure	0		0		1/2	50.0%	1/1	100%	1/4	25.0%
Hypercholesterolemia	3/4	75.0%	2/2	100%	1/2	50.0%	1/1	100%	2/4	50.0%
Stroke	0		0		0		0		0	
Heart attack	0		0		0		0		0	
Current nicotine use	0		0		0		0		0	

Abbreviation: DM, diabetes mellitus.

Patients with an HbA1c value in the prediabetic range were offered an OGTT, of whom 11 of 51 (21.6%) underwent the test. The results, including the Matsuda score, are listed in Table S1 [22].

The median plasma glucose levels were 101 (75-114) mg/dL, 135 (78-192) mg/dL, and 99 (49-167) mg/dL at 0, 60, and 120 minutes, respectively. Median insulin levels at these time points were 8.1 (2.8-36.3) mIU/L, 101 (10-279) mIU/L, and 50 mIU/L. The median Matsuda index was pathological, with 3.8 (0.9-10.2) revealing impaired insulin sensitivity in this cohort. Six of 11 (54.6%) survivors presented with a pathological Matsuda score <4 and 2 (18.2%) with a score between 4 and 6. Only 3 (27.3%) of these survivors showed preserved insulin sensitivity. No diagnosis of DM was established based on the OGTT. Data are presented as median (range).

In the ANOVA, mean HbA1c levels differed across the 5 radiation groups (*P* = 1.38 × 10^−5^), whereas random glucose levels did not show a difference (*P* = .254). In the ANCOVA adjusting for BMI, the association between radiation group and HbA1c shows a difference (*P* = 1.03 × 10^−5^), whereas BMI was not showing a difference with HbA1c (*P* = .216). In the ANCOVA for random glucose, the association with radiation group did not show a difference (*P* = .06), whereas BMI showed a difference with random glucose levels (*P* = 1.08 × 10^−9^). In the adjusted logistic regression model, none of the radiation groups or steroid categories showed a difference. The TBI group showed a trend toward higher dysglycemia risk compared with the reference, and steroid use also showed no difference. The results are presented in detail in Table S2 [22].

## Discussion

Advances in diagnostics and treatment have led to a global increase in the number of long-term cancer survivors. Many of them suffer from therapy-related sequelae, including metabolic disorders [23, 24]. CAYA cancer survivors with DM have a 3-fold increased risk of cardiovascular diseases and an over 4-fold increased risk of heart failure compared to those survivors without DM [15]. Insulin resistance and increasing BMI contribute to this risk [25]. An effect modification between cancer and BMI on insulin sensitivity suggests that cancer-specific factors contribute to glucose dysregulation. This could partially explain the higher incidence of DM diagnoses among cancer survivors. Additionally, they carry a high lifetime risk for further DM-associated complications. Therefore, the identification of risk groups is essential to establish specific, risk-adapted surveillance.

Traditional risk factors for DM reported in the literature include abdominal, cranial, and TBI as well as overweight among CAYA cancer survivors. Six of 197 survivors in our study (HIT-MED group) reported having DM, half of whom reported having a first-degree relative with DM, increasing their individual risk to be affected by this disease. Patients in the subgroup with DM were also all obese resulting in a weight-related higher risk to develop DM (median BMI 23.42 kg/m^2^ in the total cohort, 36.86 kg/m^2^ in the subgroup). No significant difference in DM prevalence compared to an age-matched control group (general population) could be demonstrated [17]. However, this might underestimate the true life-long risk as median age in the HIT study group was 27.1 ± 4.5 years and, as the DM risk further increases with time.

In the CLI group, DM prevalence of 7.7% in patients who underwent CSI was increased compared to those who received another kind of localized radiotherapy or no radiotherapy. Notably, a relevant part of 26.9% of patients treated with CSI was affected by prediabetic glucose levels, resulting in a total of 34.6% of CAYA survivors with a medium age of 38 years treated with CSI experiencing dysglycemia.

Although the prevalence of dysglycemia was descriptively higher in the CSI group, this association could not be confirmed in a direct statistical comparison using the Fishers exact test with the rest of the radiotherapy group. Although the observed distribution suggests a possible association between CSI and an increased metabolic risk, the lack of statistical significance may be partly explained by the limited sample size of the CSI subgroup, resulting in reduced statistical power.

A recent study by Bolier et al support our results. They demonstrated an elevated risk of DM that was nearly 3 times higher compared to the general population among survivors exposed to abdominal or pelvic radiotherapy. Cancer survivors treated with TBI had an even higher risk, which is in line with our results. They also included a small group of central nervous system tumor survivors (9.1%) but did not provide a separate analysis for this subgroup. Mean age of their study population was comparable to the mean age in our study cohort HIT and they presented a similar DM prevalence of 3.8% in an analysis of 2338 survivors (3.05% in our HIT cohort) by integrating clinical data from hospital records with patient-reported outcomes. Including increasing age (>35 years), elevated BMI, a family history of DM, hypogonadism, hypertension, and dyslipidemia [26].

Previous studies have demonstrated that exposure to radiation affecting the hypothalamic-pituitary axis can result in endocrine dysfunction, such as a GH deficiency and impaired insulin sensitivity. These factors are linked to increased visceral adiposity, dyslipidemia, and insulin resistance, which may contribute to the development of prediabetes and DM [27]. However, our study provides additional insights by demonstrating that not only overt DM but also prediabetic conditions are more common in patients who are treated with craniospinal irradiation. Our analysis of metabolic parameters supports this assumption, as the ANOVA and ANCOVA models for HbA1c showed significant group effects, indicating an association between the radiotherapy exposure and long-term dysglycemia, whereas BMI association showed no significance.

A cohort study by Khanna et al discovered a significantly increased risk for all subtypes of cardiovascular diseases—including coronary heart disease, myocardial infarction, and cerebrovascular events such as stroke—in cancer survivors with DM, who exhibited a 3-fold higher risk of developing cardiovascular disease and a 4-fold higher risk of heart failure compared to survivors without DM [15]. This highlights the need for systematic long-term metabolic screening and the clinical relevance of early and comprehensive cardiometabolic monitoring in these patient groups.

In the CLI study group, almost one third of the survivors treated with CSI were at least 35 years old and more than half of these patients had an elevated BMI. Family history of DM and the presence of hypogonadism were not assessed in the CLI study; therefore, it appears that some of these risk factors are present in the CSI group suggesting an increased metabolic risk. These results highlight the increased risk of DM among CAYA cancer survivors and emphasize the need to include treatment- and patient-related factors in long-term follow-up care, as supported by the guideline from Van den Oever et al on preventing metabolic syndrome [28].

### Limitations

This study's major strengths are the different approaches to assess DM risk in CAYA brain tumor survivors using 2 different study groups. The HIT-MED group was assessed with self-reported data from questionnaires and collects patient-reported outcomes and subjective experiences, whereas the CLI group is based on documented medical records and clinically verified findings. The combination of these various approaches results in a more comprehensive and nuanced assessment of the risks and impairments among CAYA cancer survivors.

Also, the inclusion of multiple irradiation groups in the CLI group may allow direct comparison between established and new risk constellations and thus offers a better insight of how different radiotherapeutic exposures contribute to metabolic dysfunction. Moreover, the presence of metabolic parameters like random blood glucose levels and HbA1c values would allow a more accurate evaluation of glucose metabolism risk than studies solely relying on self-reported diagnoses. However, because of the limited number of cases within the subgroups, no multivariate analyses were performed, which limits the ability to further investigate potential associations. In addition, no adjustment for multiple testing was applied; therefore, the reported *P* values should be interpreted with caution.

However, there are also some limitations that need to considered. First, we observed a low response rate in the HIT-MED group study, potentially resulting in selection bias. Moreover, information on prediabetic state was not available for the HIT group. Another limitation is that in the HIT-MED group field-specific spinal radiation doses were not consistently available and only the total radiation dose was documented. Because retrospective dose reconstruction would require strong assumptions and could introduce exposure misclassification, we did not perform additional analyses.

In the CLI cohort, dysglycemia was defined solely based on HbA1c measurements, which may have led to an underestimation of the true prevalence of glucose metabolism disorders. Previous studies in CAYA cancer survivors have shown that HbA1c has limited sensitivity for the Detection of impaired glucose tolerance compared to the oral glucose tolerance test, especially in cases of isolated postprandial dysglycemia, which appears to be common among this group [29]. Another study determined that HbA1c is influenced by red blood cell turnover and glycation rates, which can differ between ethnic groups. This suggests that standard cutoffs may not be universally applicable [30]. Population-specific factors should be considered when interpreting HbA1c results in CAYA cancer survivors to avoid underestimating dysglycemia risk.

Another limitation of this study is the relatively low participation rate in the OGTT, which was most likely driven by logistical constraints (eg, scheduling, travel requirements) and could introduce selection bias.

Unfortunately, because of the different sizes of the cohort and the subgroup of CAYA cancer survivors with DM, it was not possible to make any comparable calculations with regard to the general population for the HIT study group.

In both groups, we did not have detailed information on lifestyle factors such as diet and exercise, which are known to affect metabolic health [15]. Moreover, survivors included in the CLI study experienced different chemotherapy therapies, which could also affect metabolic outcomes. Previous studies indicated that certain chemotherapeutic agents, such as glucocorticoids or asparaginase, induce hyperglycemia and may contribute to insulin resistance and an increased risk of DM because of possible injury of pancreatic β cells [5]. In this context, exposure to chemotherapy potentially affecting glucose metabolism was presumably highest in the CLI cranial radiation and TBI subgroups, which include leukemia survivors treated with glucocorticoids and asparaginase. However, as chemotherapy for medulloblastoma generally does not include agents known to worsen glucose metabolism, being primarily based on alkylating agents, platinum compounds, and vinca alkaloids. Therefore, in our view, this effect does not substantially impact the main finding of our study that brain tumor survivors treated with CSI may be more frequently affected by dysglycemia than previously recognized. Future prospective studies with standardized treatment data are necessary to clarify these interactions.

## Conclusion

In this study, we observed a descriptively higher prevalence of dysglycemia in brain tumor survivors following craniospinal radiotherapy. Although the observed distribution suggests that CSI may be associated with an increased risk for dysglycemia compared to survivors who underwent other forms of radiotherapy or no radiotherapy, this association could not be confirmed statistically, likely because of limited sample size. Therefore, this should be interpreted with caution. Nevertheless, these findings underline the importance of metabolic monitoring in survivorship care, especially for those receiving multimodal treatment for central nervous system tumors. Additional studies are needed to verify these results as many potential confounders need to be taking into account. Prediabetic state represents a potentially modifiable risk factor that should be targeted to reduce comorbidities and future health impairment. Promotion of a healthy lifestyle in these high-risk groups as part of regular LTFU care could avoid obesity and metabolic syndrome.

## Data Availability

Some or all datasets generated during and/or analyzed during the current study are not publicly available but are available from the corresponding author on reasonable request.

## Abbreviations

ANCOVA: analysis of covariance
BMI: body mass index
CAYA: childhood, adolescent, and young adult
CSI: craniospinal irradiation
DM: diabetes mellitus
HbA1c: hemoglobin A1c
LTFU: long-term follow-up
OGTT: oral glucose tolerance test
TBI: total body irradiation
